# Effect of intermittent fasting 5:2 on body composition and nutritional intake among employees with obesity in Jakarta: a randomized clinical trial

**DOI:** 10.1186/s13104-022-06209-7

**Published:** 2022-10-12

**Authors:** Fiastuti Witjaksono, Erfi Prafiantini, Anni Rahmawati

**Affiliations:** Departement of Nutrition, Faculty of Medicine, Universitas Indonesia — Dr. Cipto Mangunkusumo General Hospital, Jakarta, Indonesia

**Keywords:** Intermittent fasting 5:2, Body composition, Fat mass, Fat-free mass, Obesity, Employees

## Abstract

**Objective:**

This study aimed to determine the effect of intermittent fasting 5:2 on body composition in employees with obesity in Jakarta.

**Results:**

Fifty participants were included; 25 were allocated to the fasting group and 25 to the control group. There was no significant change in fat mass, fat-free mass, skeletal muscle, and BMI (*p* > 0.05). Significant in-group changes were observed in body weight (*p* = 0.023) and BMI (*p* = 0.018) in the fasting group. Dietary intake was similar before and during the intervention. The reduction in macronutrient intake resulted in a statistically significant difference in carbohydrate, protein, and fat intake in the two groups (*p* < 0.05). Intermittent fasting 5:2 results in weight loss but does not affect fat mass and fat-free mass reductions. None of the between-group differences were clinically relevant.

**Trial registration:**

ClinicalTrials.gov with ID: NCT04319133 registered on 24 March 2020.

**Supplementary information:**

The online version contains supplementary material available at 10.1186/s13104-022-06209-7.

## Introduction

The incidence of noncommunicable diseases has increased worldwide. Noncommunicable diseases cause the death of 41 million people annually, accounting for 71% of all deaths globally. Obesity is a risk factor for noncommunicable diseases, such as type 2 diabetes, coronary heart disease, and metabolic syndrome. The distribution of body fat in people with obesity is significantly associated with the risk of morbidity and mortality. Adipose tissue plays a critical role in insulin resistance and obesity-related metabolic diseases [1]. Obesity should become more prevalent in Indonesia. In 2016, there were 1.9 billion overweight adults, with 650 million obese [2]. Based on the 2018 Indonesia Basic Health Research, the prevalence of obesity has increased compared to 2013, from 14.8 to 21.8%.

In Indonesia, working-age is an adult and productive age group. Obesity is most prevalent in this age group [3]. The number of workers in Jakarta increased by 4.83% in 2018 compared to 2017 [4]. This is consistent with the prevalence of obesity among company employees, especially in urban areas, which is still relatively high, at 50.6% for employees in the Bogor area and 56.3% in the Jakarta area [5, 6]. Excellent health of workers can be a determinant of high work productivity. However, when workers’ health is affected, their ability to think and do physical work might deteriorate, affecting the health condition of the workforce and, consequently, work productivity [7].

Lifestyle is one of the solutions in dealing with the problem of obesity. It involves the dietary habit of selecting various food types and physical activity levels [8, 9]. Fasting is one strategy for promoting a healthy lifestyle [10]. Intermittent fasting (IF) is the practice of eating and drinking only during regular intervals of time when there is a very low or no caloric intake, it is a techniques for energy restriction [11]. Time-restricted eating/feeding (TRE/TRF) is a type of intermittent fasting that is recommended to practice daily. TRE is a dietary strategy that combines total calories consumed throughout the course of 6 to 10 h during the day’s active period [12].There haven’t been many long-term clinical trials, but the ones that have shown that intermittent fasting (IF) is superior to caloric restriction in reducing waist circumference and central fat distribution—both of which are beneficial because these parameters are essential in reducing cardiovascular risk [13, 14]. Similarly, preliminary human research indicate that TRF enhances clinical outcomes, including body weight, blood pressure, and insulin sensitivity, at least when dietary intake is restricted to the morning or afternoon. The circadian system can explain these effects associated to the time of day since eating in accordance with our metabolism’s circadian cycles can improve cardiometabolic health [15, 16]. Bad mood, slight dizziness, weariness, headaches (which are alleviated by drinking water), and headaches are some negative effects of fasting [17].

The global rate of obesity is significantly rising, including in Indonesia. Obesity can lead to non-communicable diseases and metabolic disorders. [1, 18] The effectiveness of intermittent fasting as a method of reducing obesity varies depending on the population studied.[19, 20] Therefore, research on the effects of intermittent fasting on body composition is necessary because of contradictory outcome and protocol difference of intermittent fasting, especially in company employees with obesity. Based on this background, the purpose of this study was to compare the body composition of obese employees in Jakarta on fasting days (at dawn and iftar) and non-fasting days to determine the impact of fasting for two days per week without calorie restriction.

## Main text

## Methods

### Design

A non-blinded randomized controlled trial with 1:1 ratio two arm was used in this investigation to compare a control group that did not fast at all with an intervention group that fasted twice a week. This study has received approval by the Health Research Ethics Committee of Faculty of Medicine, Universitas Indonesia — Dr. Cipto Mangunkusumo General Hospital with ethical standards of KET-281/UN2.F1/ETIK/PPM.00.02/2020. This study has also been registered at clinicaltrial.gov with ID: NCT04319133. The study adheres to the CONSORT Guidelines.

### Settings and participants

This study was conducted at selected companies in Jakarta from July to September 2020. The screening and filling out a questionnaire attended by 67 male employees in two companies was the first step in the subject selection. The screening included anthropometry and screening interviews using a questionnaire. There were 50 subjects willing to participate in the entire study series, divided into two groups: intervention and control. The questionnaire was distributed purposively to prospective subjects who satisfied the inclusion criteria; men aged 19–59 years, with a body mass index (BMI) ≥ 25 kg/m^2^, a waist circumference of 90 cm, and fasting blood glucose < 125 mg/dL. Exclusion criteria included subjects who had participated in a weight loss program, either by diet or exercise, and who had obesity with type 2 diabetes, as evidenced by fasting blood sugar assessment. Subjects selected to participate in the study were willing to fill out an informed consent form. Drop-out criteria include subjects who refuse to have blood drawn during the baseline and endline data collection, research subjects who refuse or are unable to continue the study, and subjects who do not fast more than four times.

### Randomization

This study uses an open trial and randomizes participants through www.randomlists.com. Simple randomization processes (computerized random numbers) were used to allocate participants to one of two treatment groups at random.

### Sample size

The sample size was determined using the formula for the difference in the mean of the two populations in independent sample,[21] with a confidence interval of (1 -) 90% and power (1 -) 80%.

### Data collection

Before the study, data on food intake were obtained using food recall 2 × 24 h, nonconsecutive, consisting of one weekday (as a working day) and one weekend (as a day not working). Researchers and enumerators conducted food recall interviews. Food and drink consumption at suhoor and iftar for the fasting group was also recorded. The recording was done for 16 days of fasting (32 times of recording) through the food record method. The age of the subject was calculated based on the number of years from the year of birth to the last birthday, that proved by identity card. Interview data characteristics included education, income, knowledge, physical activity, and history of food intake.

We classified education based on the Law of the Republic of Indonesia Number 20 of 2003 about the National Education System. Low, if the subject is illiterate, does not attend school, has finished or did not finish elementary school (SD), junior high school (SLTP), did not finish high school (SLTA), or the equivalent. Moderate, if the subject has graduated from high school or equivalent, but has not graduated from an academy or college. High, if the subject has graduated from an academy or college [22]. Research subjects were grouped based on the amount of income they received in one month, classified based on the regional minimum wage (UMR) in DKI Jakarta. Less, if income < 3.940.000 IDR per month, and enough, if income ≥ 3.940.000 IDR per month [23].

The level of nutritional knowledge is determined based on the answer to the questionnaire and given a score. If the answer is wrong or does not know, it is given a value of 0. If it is correct, it is given a value of 1. In the scoring system, the correct number is divided by the number of questions multiplied by 100% and then categorized into: less if the value is less than 60%, medium, if the value is between 60% and 80%, and excellent if the score is greater than 80% [24, 25].

Physical activity categories based on the International Physical Activity Questionnaires (IPAQ). Light physical activity: does not meet the requirements for moderate-heavy activity or activities with an intensity of 600 METS minutes per week. Moderate physical activity: 3 days of vigorous physical activity for at least 20 min per day, 5 days of moderate physical activity/walking for at least 30 min per day, or 5 days of a combination of walking, moderate activity, and vigorous activity for at least 600 METS minutes per week. Heavy physical activity: 3 days of vigorous activity with a total of at least 1500 METS minutes per week or 7 days of walking, moderate activity, and vigorous activity with a total of 3000 METS minutes per week [26, 27].

Bodyweight and body composition, including percent body fat, muscle, and fat mass, were measured using a bio-impedance analyzer (Tanita 541) after overnight fasting, including water (> 12 h). Height was measured using a shorrboard. According to the Asian-Pacific cutoff points, BMI is computed as weight in kilograms divided by the square of height in meters (kg/m2) and is classified into into : underweight (< 18.5 kg/m^2^), normal weight (18.5–22.9 kg/m^2^), overweight (23–24.9 kg/m^2^), obese 1 (25–29,9 kg/m^2^), and obese 2 ≥ 29,9 kg/m^2^) [28].

Trained enumerators assessed all measurements, and a similar enumerator performed each measurement. All post intervention study variables were measured on Saturday, which was the day after the end of the eighth week of intervention.

### Intervention

This research was divided into three phases: the baseline, intervention, and endline phases. In the baseline phase, the subject was interviewed, had body measurements taken, and underwent laboratory examinations.

Furthermore, the subject had body measurements, including weight and height measurements. Both the intervention and control groups were educated about balanced nutrition and information about obesity. Education was provided once with presentation media via Power Point and continued with discussion. Through educational intervention, subjects would have a better understanding and more awareness of nutritional knowledge reflected in changes in dietary habits during the study.

The fasting method was modified two days a week, on Mondays and Thursdays, for eight weeks in the intervention phase. Fasting cannot occur on days other than Monday and Thursday. Fasting is done for ± 14 h, starting from sunrise to sunset or from 04:00 a.m. to 06:00 p.m. There is no restriction on calorie intake at dawn and iftar. Researchers will compare perceptions regarding the implementation of fasting with the intervention group. Fasting groups would be reminded to fast via Whatsapp every Monday and Thursday night. The weekly monitoring form and the outcomes of dietary records reveal fasting subject’s compliance. Two months later, all subjects had finished reporting on food diary and they were invited to do an end-line body composition test.

### Data Analysis

The data distribution was examined using univariate analysis in this study (as descriptive data). To analyze the mean fat mass and fat-free mass before and after treatment, the bivariate analysis used paired t-test if the distribution was normal or Wilcoxon if the distribution was not normal. Unpaired t-test was used if the distribution was normal or Mann–Whitney if the distribution was not normal to analyze the difference in mean changes in fat mass and fat-free mass after treatment between the treatment and control groups. The data is considered significant if the *p*-value is < 0.05.

## Results

Among 67 participants, three participants who did not satisfy inclusion criteria and 12 who were unwilling to join the study were excluded. All participants were randomly allocated into two groups (fasting = 26, control = 26). Two subjects was lost to follow up; and consequently 25 patients for each groups remained for the per-protocol analyses. Supplementary file Figure S1 depicts the flow of participants in the study process.

### Baseline characteristics

Table 1 shows the baseline characteristics of all study participants. No significant differences in demographic characteristics were observed between both groups (*p* ≥ 0.05).

**Table 1:** Baseline characteristics

Characteristic	Group			*p*-value
	**Intervention** **(n = 25)**	**Control** **(n = 25)**		
Age, years	32 (19–52)		30 (22–54)	0.763^1^
Nutritional status, n(%)				
Obesity I	10 (40)		11 (44)	0.774^2^
Obesity II	15 (60)		14 (56)	
Education, n(%)				
Moderate	7 (28)		7 (28)	> 0.999^2^
High	18 (72)		18 (72)	
Income, n(%)				
Less	3 (12)		6 (24)	0.269^2^
Enough	22 (88)		19 (76)	
Knowledge, n(%)				
Less	4 (16)		3 (12)	0.916^2^
Moderate	15 (60)		16 (64)	
Good	6 (24)		6 (24)	
IPAQ score, n(%)				
Low	11 (44)		9 (36)	0.276^2^
Moderate	9 (36)		14 (56)	
High	5 (20)		2 (8)	

^1^ Mann–Whitney, ^2^ Chi-Square

Prior to the study, there was no significant difference in the intake of the two groups based on food intake data. However, there were significant differences in total calories, carbohydrate, protein, and fat intake between the intervention and control groups after the intervention (*p* < 0.05). In the intervention group, food intake during the study was obtained by recording on fasting days only. The average intake of the intervention group was lower than that of the control group. Table 2 provides detailed results. Table 3 shows participants’ body weight and body composition at baseline, post-intervention, and values of change.

**Table 2:** Intake of energy and macronutrients before and during the intervention

Variable	Group		*p*-value
	**Intervention (n = 25)**	**Control (n = 25)**	
Energy intake (kcal/day)			
Before	1615.56 ± 540.94	1578.34 ± 393.87	0.782^1^
During intervention	897.05 (505.45–1344.25)	1357.35 (871.2–2946.72)	< 0.001 ^2^
Changes	−628 ± 526.81	−154.73 ± 576.56	0.004^1^
Carbohydrate (g/day)			
Before	203.7 (74.85–296.2)	179.7 (120.9–355.05)	0.393^2^
During intervention	122.63 (60.59–178.35)	161.41 (93.72–328.11)	< 0.001 ^2^
Changes	−81.55 ± 51.97	−18.40 ± 56.52	< 0.001^t^
Protein (g/day)			
Before	55.79 (18.3–132.5)	57.05 (23.45–108.25)	0.961^2^
During intervention	36.88 (19.72–58.20)	48.90 (30.73–220.49)	0.001^2^
Changes	−18. 10 (− 95.48–19.74)	−7.81 (− 67.57–48.04)	0.017^2^
Fat (g/day)			
Before	63.08 ± 29.93	64.89 ± 29.08	0.828^1^
During intervention	36.73 (19.31–60.14)	57.28 (16.83–138.3)	0.001^2^
Changes	−24.79 ± 30.60	−7.18 ± 38.53	0.08^1^

^1^ Independent t-test, ^2^ Mann–Whitney

**Table 3:** Changes in body weight and body composition

Parameter	Group		*p*-value
	**Intervention (n = 25)**	**Control (n = 25)**	
Fat mass (%)			
Before	29.74 ± 4.19	29.49 ± 4.62	0.846^1^
After	29.45 ± 4.25	29.30 ± 4.49	0.903^1^
Fat mass changes	0.000 (− 2.5–1.7)	−1.11 (− 3.1–1)	0.527^2^
Fat-free mass (%)			
Before	63.08 ± 5.72	62.73 ± 6.21	0.836^1^
After	62.82 ± 5.71	62.9 ± 6.4	0.956^1^
Fat free mass changes	−0.2 (− 1.9–2)	−0.3 (− 2.9–9.3)	0.484^2^
Skeletal muscle (kg)			
Before	59.83 ± 5.43	59.53 ± 5.85	0.852^1^
After	59.58 ± 5.42	59.66 ± 6.09	0.963^1^
Skeletal muscle changes	−0.2 (− 1.8–1.9)	−0.3 (− 2.8 − 8.9)	0.56^2^
Visceral fat rating			
Before	15 (10–20)	14 (11–21)	0.577^2^
After	15 (10–20)	14 (11–21)	0.666^2^
Visceral fat rating changes	0.000 (− 2–1)	0.000 (− 2–1)	0.264^2^
Body weight (kg)			
Before	90.54 ± 13.35	89.77 ± 12.71	0.835^1^
After	89.76 ± 13.2	89.24 ± 12.67	0.888^1^
Body weight changes	−0.8 (− 5.1–2.2)	−0.3 (− 7.9–2.8)	0.420^2^
BMI (kg/m^2^)			
Before	30.8 (26.7–43)	30.4 (25–44.1)	0.831^2^
After	30.3 (27.10–42)	29.9 (25.7–43. 5)	0.961^2^
BMI changes	−0.3 (− 2–0.7)	−0.1 (− 2.7–1)	0.302^2^

^1^ Independent t-test, ^2^ Mann–Whitney

### After intervention

The intervention group’s macronutrients consumed by research subjects were 50.4% carbohydrates, 13.6% protein, and 35% fat. Meanwhile, the macronutrient consumed by the control group was 45.5% carbohydrates, 14.5% protein, and 37% fat. We have conducted a Wilcoxon analysis to see the difference before and after the study in the intervention group and the control group. There was a significant difference in the intervention group in the intake of energy, carbohydrates, protein, and fat in the measurements before and during the intervention, with p < 0.05. While in the control group, there was no significant difference in the control group in the intake of energy, carbohydrates, protein, and fat in the measurements before and during the intervention, with p > 0.05.

In body composition, there was no significant difference in fat mass, skeletal muscle, and visceral fat rating before and after the study, both in the intervention group and in the control group. Meanwhile, fat free mass before and after the study experienced a significant difference, with p = 0.05 in both groups. Body weight and body mass index in the intervention group had a significant difference before and after the study with p = 0.023 and p = 0.018, respectively. While in the control group, body weight and body mass index did not experience a significant difference before and after the study.

## Discussion

Among the 52 participants, 50 of them completed the trial. Two participants dropped out because they resigned from work and were out of contact before the post-assessment. Individuals with obesity need to maintain or increase lean body mass during weight reductions because it directly affects one’s resting metabolic rate and energy expenditure, contributing to substantial weight loss.

The recommended carbohydrate composition is 50–60%, 15% protein consumption, and 25% fat consumption based on balanced nutrition guidelines. Based on the data, there is a gap between the food intake of the research subjects and the recommendations from the balanced nutrition guidelines [29]. The level of fat consumption in research subjects was higher than the recommendation, which was 35% of total energy intake.

It is expected that food consumption will be lower during a fast, since two meals are often eaten between sunset and dawn and because of the changing meal timings while fasting [30]. During the 8-week treatment period, there was a decrease in macronutrient intake in both groups. The reduction in macronutrient intake resulted in a statistically significant difference in carbohydrate, protein, and fat intake in the two groups (*p* < 0.05). The food intake results during the intervention phase were obtained from food records. The recording was performed on fasting days only in the intervention group, while food intake on non-fasting days was not recorded. The recording was performed three times on a nonconsecutive day in the control group. Meanwhile, body weight, body fat, and other factors did not decrease when the study participants were fasting. During fasting, it appears that body mass, fat, and lean—are all very well maintained regardless of the subject’s caloric expenditure.

During the 8-week treatment period, there was a decrease in macronutrient intake in both groups. The reduction in macronutrient intake resulted in a statistically significant difference in carbohydrate, protein, and fat intake in the two groups (*p* < 0.05). The food intake results during the intervention phase were obtained from food records. The recording was performed on fasting days only in the intervention group, while food intake on non-fasting days was not recorded. The recording was performed three times on a nonconsecutive day in the control group.

In this study, energy restriction was not implemented in either the intervention or control groups. However, participants in those groups were educated about obesity and instructed to consume food according to balanced nutrition guidelines at the beginning of the intervention. Participants in the intervention group lost 0.8 kg, and participants in the control group lost 0.3 kg after eight weeks of intervention. Furthermore, BMI decreased by 0.3 kg/m^2^ in the intervention group and by 0.1 kg/m^2^ (*p* < 0.05) in the control group. Although there was a statistically significant association between body weight and body mass index in the intervention group before and after the study, this association is likely not clinically relevant, as it is less than 1 kg during 8 weeks intervention. There was no significant difference in muscle mass, fat mass, and percent body fat between the two groups. The study revealed that fat concentrations could occur if weight loss exceeds 5% of initial body weight [31], but the weight loss in this study was only 0.92%.

After following a weight-loss intervention protocol, a recent study found that late eaters had a lower weekly weight-loss rate than early eaters [32]. We suggest this mechanism similar to our finding. During a fast, eating habits can change. Some participants eat suhoor between three and five in the morning, while others take their final meal before bedtime at around midnight (23:00). The circadian rhythm may be responsible for this discrepancy in mealtimes. Our “biological clock,“ the suprachiasmatic nucleus in the hypothalamus, controls the circadian rhythm in animals [33]. Internal oscillators’ modulation of circadian rhythms is crucial for metabolic regulation. Every species has a tendency to eat when it is active, whether that is day or night depending on the species. Circadian rhythm is biologically regulated, and many animals’ studies demonstrate metabolic disarrangements when food intake occurs in the rest phase of the species [34, 35].

Different research results are shown by Fudla et al. [36]. The study discovered a significant change in terms of BMI between the control and intervention groups in only four weeks (*p <* 0.05). The participants in the control group experienced a slightly decreased BMI, whereas the intervention group’s BMI decreased significantly. Since the physical activity data show that the majority of the subjects had moderate IPAQ scores, same like in our study, so may the causes for the contradictory results about the effects of fasting is because study groups used. The average age of the subjects in this study was 32 years, with a maximum age of 54 years. In contrast, the research subjects in Fudla’s study were on average about 19 years old [36]. However, the mentioned trials participants were younger, which may explain the weight loss differences compared to our study. Ever since the age of 25, weight gain is due to a gain an increase in body fat and a loss of lean muscle. The causes of decreasing fat-free mass are thought to be decreased body cell mass owing to aging and decreased protein synthesis due to lower anabolic hormone concentrations [37, 38]. Age-related reductions in resting energy expenditure (REE) range from 1 to 2% every decade [39]. Decreases in REE and body composition started between 30 and 45 years [40]. This age gap will have an impact on basal metabolic rate (BMR), which may cause older age groups to lose weight more slowly than young adults.

## Conclusion

The reduction in macronutrient intake resulted in a statistically significant difference in carbohydrate, protein, and fat intake in the two groups (*p* < 0.05). Meanwhile, there was no significant difference in the mean change in fat mass, muscle mass, and percentage body fat in the fasting group compared to the non-fasting group during the 8-week intervention period. However, there was a difference in body weight in the intervention group.

## Limitation

This randomized controlled clinical trial study has several limitations. First, measurement error causes inaccurate food recording (self-administered) so that there is a possibility of under reporting. Second, the study used a bioimpedance analyzer to measure the body composition of study participants. The bio-impedance analyzer for body composition measures should be considered a limitation of this study because the dual-energy absorptiometry technique is the gold standard for measuring body composition in clinical studies. Third, failure to evaluate the intervention group’s food intake on days when they weren’t fasting and consider its possible effects on body composition in this study.

## Electronic supplementary material

Below is the link to the electronic supplementary material.

Supplementary Material 1

## Data Availability

The data used during this study are not publicly available due to the informed consent and ethics approval not containing approval from the participants for data sharing. Reasonable requests would be considered in consultation with the of Faculty of Medicine, Universitas Indonesia—Dr. Cipto Mangunkusumo General Hospital.

## Abbreviations

BMI: Body Mass Index.
BMR: Basal Metabolic Rate.
IPAQ: International Physical Activity Questionnaire.
