# Supplementary material for: Effect of intermittent fasting 5:2 on body composition and nutritional intake among employees with obesity in Jakarta: a randomized clinical trial
Source: BMC Res Notes. 2022 Oct 12;15:323. doi: 10.1186/s13104-022-06209-7 (PMC9559012; doi:10.1186/s13104-022-06209-7)
Supplement: Supplementary file 1 — Supplementary Material 1 [file 13104_2022_6209_MOESM1_ESM.docx]

**Figure S1.** CONSORT Diagram

Out of contact (n=1)

Intervention group (n=26)

Control group (n=26)

Out of contact (n=1)

Completed intervention and analyzed (n=25)

Completed intervention and analyzed (n=25)

Candidates (n=330)

Excluded (n=15)

Abnormal fasting blood glucose (n=3)

Declined to participate (n=12)

Randomized (n=52)

Total screening of obese employees (n=67)
